# Rescuing the Corticostriatal Synaptic Disconnection in the R6/2 Mouse Model of Huntington’s Disease: Exercise, Adenosine Receptors and Ampakines

**DOI:** 10.1371/currents.RRN1182

**Published:** 2010-09-20

**Authors:** Carlos Cepeda, Damian M. Cummings, Miriam A. Hickey, Max Kleiman-Weiner, Jane Y. Chen, Joseph B. Watson, Michael S. Levine

**Affiliations:** ^*^Intellectual and Developmental Disabilities Research Center, David Geffen School of Medicine, University of California, Los Angeles, CA, USA and ^‡^Department of Neurology, David Geffen School of Medicine, University of California, Los Angeles, CA, USA.

## Abstract

In the R6/2 mouse model of Huntington’s disease (HD) we examined the effects of a number of behavioral and pharmacological manipulations aimed at rescuing the progressive loss of synaptic communication between cerebral cortex and striatum. Two cohorts of transgenic mice with ~110 and 210 CAG repeats were utilized. Exercise prevented the reduction in striatal medium-sized spiny neuron membrane capacitance but did not reestablish synaptic communication. Activation of adenosine A2A type receptors renormalized postsynaptic activity to some extent. Finally, the ampakine Cx614, which has been shown to prevent α-amino-3-hydroxyl-5-methyl-4-isoxazole-propionate (AMPA) receptor desensitization, slow deactivation, and facilitate glutamate release, induced significant increases in synaptic activity, albeit the effect was somewhat reduced in fully symptomatic, compared to control mice. With some limitations, each of these strategies can be used to delay and partially rescue phenotypic progression of HD in this model.

**Introduction**  

Huntington’s disease (HD) is an insidious, progressive and fatal neurodegenerative disorder caused by a mutation that expands the number of CAG (glutamine) repeats [1]. Neuropathologically, HD is characterized by loss of striatal medium-sized spiny neurons (MSSNs), as well as more discrete cell loss in other brain areas [2]. The symptoms include motor abnormalities, the more prominent being chorea, as well as cognitive and psychiatric disturbances [3]. 

            Genetic mouse models of HD recapitulate some, but not all, of the phenotypic alterations found in human HD [4]. In particular, the R6/2 model with ~150 CAG repeats has provided a wealth of information about mechanisms of disease progression and represents a useful tool for drug screening as the progression of the phenotype occurs rapidly [5] and motor, histopathological, and neuronal functional changes are reliable markers of the disease. 

            Our laboratory has characterized a number of morphological and electrophysiological alterations in striatum and cortex of these mice [6] [7]. We demonstrated reduced membrane capacitance and increased input resistance in neurons from symptomatic R6/2 mice, along with decreases in somatic size, dendritic field, and number of spines of striatal MSSNs and cortical pyramidal neurons [8]. 

            In addition, synaptic changes along the corticostriatal pathway occur [8] [9] [10]. An early and a late synaptic phenotype can be characterized in striatal MSSNs. Early, synaptic dysregulation of glutamate release is manifested by the occurrence of large-amplitude spontaneous excitatory postsynaptic currents (EPSCs) that reflect cortical hyperexcitability. Such increased excitability could explain the propensity to seize and the lower convulsive threshold to systemic administration of epileptogenic agents in R6/2 mice [11]. Large synaptic events in MSSNs can be reduced by riluzole and valproic acid [9] and cortical hyperexcitability can be renormalized by anticonvulsant agents such as rolipram and tiagabine [12] [13]. Similarly, reducing glutamatergic transmission by decortication around 4-6 weeks of age can be beneficial in R6/2 mice [14]. However, at about 5-7 weeks of age, a progressive reduction in the frequency of spontaneous EPSCs in MSSNs occurs, leading to a virtual disconnection between cortex and striatum [9]. This disconnection is deleterious as it deprives the striatum of essential trophic factors produced by cortical pyramidal neurons, such as brain-derived neurotrophic factor (BDNF), and could also result in a deficit in survival signaling by synaptic N-methyl-D-aspartate (NMDA) receptors and preferential activation of proapoptotic extrasynaptic receptors [15] [16] [17] [18].

            As the progressive disconnection between cortex and striatum could explain many of the motor and cognitive symptoms, re-establishing normal connectivity (i.e., increasing glutamatergic synaptic transmission), may be helpful to ameliorate or reduce progression of the disorder. The present series of studies used behavioral and pharmacological manipulations known to be effective in slowing the progression of symptoms in HD and other neurodegenerative disorders, in attempts to slow or rescue the late synaptic phenotype.

            The beneficial effect of voluntary exercise in Parkinson’s (PD) and Alzheimer’s (AD) diseases has been well documented [19]. Exercise increases BDNF content and neurogenesis [20] [21] [22], which could explain its benefits. However, there is little information on the effects of exercise *per se* on the progression of symptoms of HD and, to our knowledge, no data on the effects of exercise on synaptic activity in the corticostriatal pathway. Exercise may turn out to be beneficial, as in R6/1 mice, an enriched environment delays the onset of the HD phenotype [23].

            We also targeted adenosine A_2A_ receptors as there is evidence that their modulation can ameliorate symptoms of PD [24] [25]. In the R6/2 mouse, early changes in adenosine receptors and content occur and these changes could contribute to the HD phenotype [26]. In addition, recent studies indicate that adenosine receptor agonists differentially modulate NMDA receptor-mediated excitotoxicity in R6/2 mice [27] and both A_2A_ receptor agonists and antagonists can be used to treat HD symptoms [28] [29]. However, little is known about the cellular mechanisms underlying A_2A_ receptor dysfunction in HD. Thus, we examined the effects of A_2A_ receptor modulators on spontaneous glutamate synaptic currents.

            Finally the ampakines, drugs that slow deactivation of α-amino-3-hydroxyl-5-methyl-4-isoxazole-propionate (AMPA) glutamate receptors [30], have been suggested as potential therapeutic targets in a number of neurological disorders [31]. We tested Cx614, as this compound has been shown to increase BDNF production [32] and BDNF rescues synaptic deficits in the hippocampus of a knock-in mouse model of HD [33].

**Methods**

All experimental procedures were performed in accordance with the United States Public Health Service *Guide for Care and Useof Laboratory Animals* and were approved by the Institutional AnimalCare and Use Committee at the University of California, Los Angeles (UCLA).

**Mice, Ages and Treatments: **R6/2 transgenic mice and wildtype (WT) littermates were obtained from our colony at UCLA. All animals were genotyped twice, once after weaning and again after the electrophysiological recordings. In transgenic mice the number of CAG repeats also was measured. Mice for the exercise study had 106-114 CAG repeats, while mice for the adenosine receptor study had 200-220 CAG repeats. Mice used for the ampakine study had 106-121 CAG repeats. 

**Voluntary Exercise: **Four groups of mice (including both males and females) were examined. Two groups of WT and R6/2 mice (aged 3 weeks, n=9 in each group) were placed in individual cages equipped with a running wheel as previously described [34] [35]. Two other groups of WT and R6/2 littermate mice (n=9 in each group) were placed in individual cages with running wheels but these were fixed and immobile. Briefly, for running behavior, cages were equipped with a running wheel (23 cm diameter, Mini Mitter Company Inc., Bend OR) and rotations of the wheel were detected and recorded in 3 min bin intervals (VitalView Data Acquisition Software V 4.0, Mini Mitter Company Inc., Bend OR). Wheel running activity during light and dark phases was calculated subsequently (ActiView, V 1.2, Mini Mitter Company Inc., Bend OR). After 3-6 weeks in the cages mice were sacrificed for slice electrophysiology and BDNF measurements. However, 3 animals (one WT and two transgenic) died and were not used for electrophysiological recordings.

**BDNF Determination**: We used the BDNF E_max_^® ^Immunoassay System from Promega to quantify BDNF by ELISA in mouse striatum obtained from the same groups of WT and R6/2 mice either with or without running wheel as exercise. The striatum was dissected out, flash frozen on dry ice and stored at -80^o^C until assayed. Prior to each assay, the striatum was homogenized in lysis buffer (137mM NaCl, 20mM Tris-HCL at pH 8, 1% NP40, 10% glycerol, 1mM PMSF, 10μg/ml aprotinin, 1μg/ml leupeptin, 0.5mM sodium vanadate) by combined douncing and sonication followed by microcentrifugation at 16,000 g. The supernatant fraction was saved for ELISA and its protein concentration was determined by the Bradford method [36]. A standard BDNF sample and multiple striatal supernatant samples were serially diluted in duplicate in 96-well plates and the ELISA was carried out using a combination of monoclonal and polyclonal antibodies to BDNF. Striatal BDNF values were determined by comparison to a BDNF Standard curve (0-500 pg/ml). Final BDNF concentrations in striatal supernatants were calculated as pg/µg total striatal protein.

**Adenosine A_2A_ Receptors: **Experiments were conducted in pre- (21-41 days, n=7) or symptomatic (>60 days, n=5) R6/2 mice and WT controls (n=7 and 6, respectively). In some additional experiments mice expressing enhanced green fluorescent protein (EGFP) in A_2A _or D2 receptor-containing MSSNs were used to examine adenosine receptor modulation specifically in this subpopulation of neurons that originate the indirect striatal output pathway.

**Ampakines: **Experiments were conducted in two age groups of R6/2 and WT controls: a middle-agegroup (5-7 weeks; n=12 R6/2 and 14 WT) corresponding to theonset of motor symptoms, and an older group (11-12 weeks; n=^ ^7 R6/2 and 7 WT) displaying the full behavioral phenotype. 

**Slice Electrophysiology:** After sacrifice, the brains were dissected and immediatelyplaced in oxygenated ice-cold low-Ca^2+^ artificial cerebrospinalfluid (ACSF) containing (in mM) NaCl, 130; NaH_2_PO_4_, 1.25; NaHCO_3_,26; MgCl_2_, 5; CaCl_2_, 1; and glucose, 10. The hemispheres wereseparated and 350 µm coronal slices were cut and transferredto an incubating chamber containing ACSF (with 2 mM CaCl_2_ and2 mM MgCl_2_) oxygenated with 95% O_2_-5% CO_2_ (pH 7.2–7.4,290–310 mOsm, 25±2°C). After 1 h slices wereplaced on the stage of an upright Olympus microscope (BX51),submerged in continuously flowing ACSF (~3 ml/min). Nomarski optics and infrared videomicroscopy (IR-DIC) were used to identify MSSNs in slices [37]. A_2A_ receptor expressing EGFP-positive cells were excited with UV light and visualized using fluorescence microscopy [38]. 

            Whole-cellpatch clamp recordings in voltage clamp mode were obtained fromMSSNs using an Axopatch 200B or Multiclamp 700B amplifier operated under pClamp (versions 8 and 10, respectively). MSSNs were identified by somatic size and typical basic membrane properties (input resistance, membrane capacitance,and time constant). The patch pipette (3-5 MΩ impedance) contained the following solution(in mM): 130 Cs-methanesulfonate, 10 CsCl, 4 NaCl, 1 MgCl_2_, 5^ ^MgATP, 5 EGTA, 10 HEPES, 0.5 GTP, 10 phosphocreatine, and 0.1^ ^leupeptin, pH 7.25-7.3 (osmolality, 280-290 mOsm). Series resistance was less than 25 MΩ and was compensated optimally by the automatic compensation function included in the pClamp software. 

            Spontaneous EPSCs were examined in isolation by holding the membrane at -70 mV and, in most cases, using bicuculline (BIC, 10 μM) to block spontaneous activity mediated by activation of GABA_A_ receptors.The membrane currentwas filtered at 1 kHz and sampled at 20 kHz. In some experiments, tetrodotoxin (TTX, 1 µM) was included in the extracellular solutionto isolate the events that are not dependent on presynaptic actionpotentials [miniature EPSCs (mEPSCs)]. Spontaneous synaptic events were analyzed off-line using the Mini Analysis 6.0 Program (Jaejin Software, Leonia, NJ). This software was usedto calculate EPSC frequency, amplitude for each event, and toconstruct amplitude-frequency and inter-event interval histograms. The thresholdamplitude for the detection of an event was adjusted above root-mean-square noise level (~2-3 pA) and frequencieswere expressed as number of events per second (inHz). Analysis of EPSC kinetics was done using the Mini Analysis Program. Events with peak amplitudes between 5 and 50 pA were grouped, alignedby half-rise time, and normalized by peak amplitude. In each cell, all events between5 and 50 pA were averaged to obtain rise times, decay times,and half-amplitude durations. Second-order exponentialcurves were fit with a maximum of 5000 iterations for computation of decay time.

**Drugs: **A_2A_ receptor agonists (CGS 21680) and antagonists (KW 6002) were obtained from Sigma and the Cure HD Initiative, Inc., respectively. They were applied in the bath to examine their effects on spontaneous synaptic activity. Cx614 (gift from Cortex Pharmaceutical, Irvine, CA) was dissolved in DMSO (0.1%) at a concentration of 200 mM and stored at -20°C. Before the experiments, the drug was diluted with ACSF solution to the desired concentration.

**Statistics:** Values in the figures and text are presented as means±SEs. Differences among group means were assessed with appropriate one or two-wayANOVAs followed by Fisher’s post hoc tests (for running wheel analysis) or Student’s* t *test when only two groups were compared. Differenceswere considered statistically significant if *p* < 0.05. 

**RESULTS**

**Exercise: **Both WT and R6/2 transgenic mice ran in the wheels and there were no significant differences in total average activity between WT or transgenic males and females. Previously, we demonstrated profound deficits in running wheel behavior in R6/2 mice by 4.5 weeks of age [34]. Here, mice were exposed to running wheels for a longer period of time, and from an earlier age. As expected, most running occurred during the dark cycle. As previously shown [34], during the light cycle there were no significant differences between WT and R6/2 at this age. R6/2 mice ran significantly less than WT animals during the dark cycle (Figure 1; effect of genotype F(1,16)=28.01, p<0.0001). The difference was significant from the 6^th^ day in the running wheel cage (27 days of age) and lasted throughout the duration of the experiment.

**Figure:**
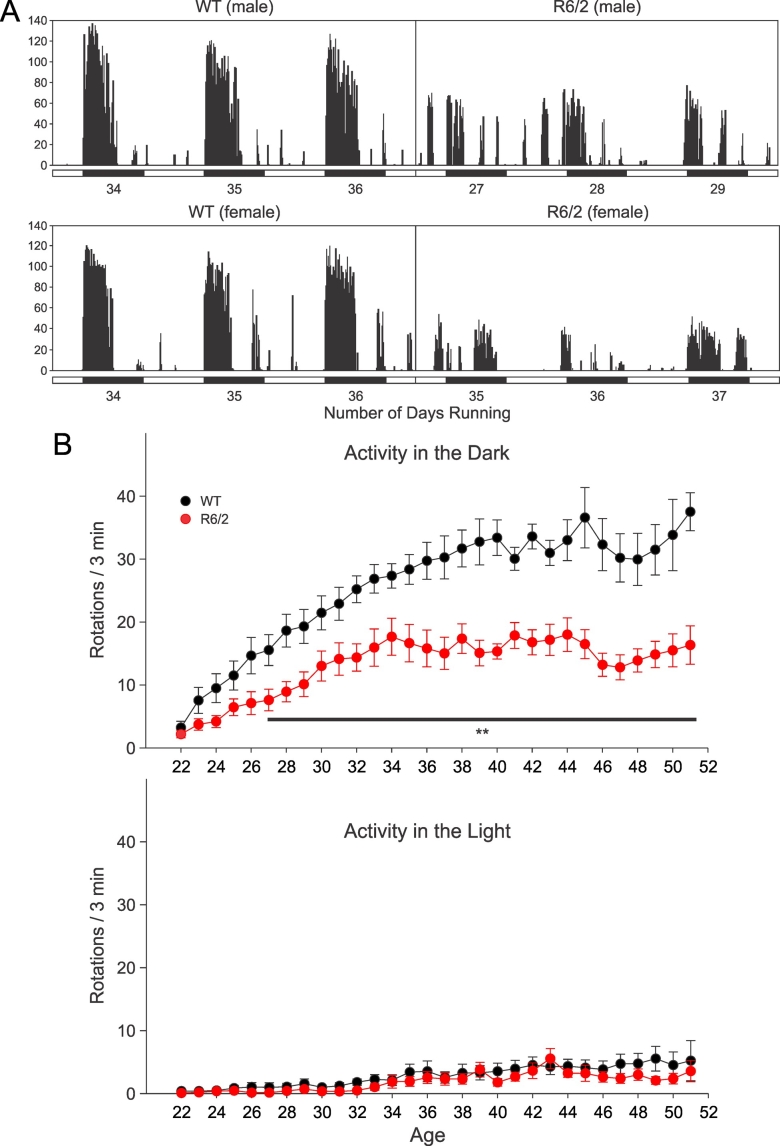

**Fig. 1: A.** Examples of actograms from male and female WT (left) and R6/2 (right) mice during the light and dark cycles. R6/2 mice displayed reduced activity compared to WTs. Each value represents the number of rotations per 3 min. The bars under the actograms show the light and dark 12 h cycles. These actograms were obtained during the 3 days preceding the electrophysiological recordings. The number of days in the running wheel cage is also indicated. **B**. Average activity during the dark and light cycles. Throughout the duration of the experiment, R6/2 mice displayed lower levels of activity than WTs in the dark. The differences were statistically significant (p<0.01) from the sixth day on (indicated by the line and asterisks). There were no significant differences during the light cycle.

MSSNs from non-running transgenic mice had reduced membrane capacitance and increased input resistance compared to WTs. These changes have been reported previously and probably indicate loss of cell membrane area [8] [9] as well as reduced density of K^+^ channels [39]. Running alleviated the significant difference in cell membrane capacitance between the groups (i.e., the difference between WT and R6/2 mice was no longer statistically significant) (Table 1). In contrast, the increase in input resistance in R6/2 mice was only slightly alleviated by exercise (i.e., the magnitude of the increase in input resistance was diminished but the difference between WT and R6/2 mice was still statistically significant) (Table 1). The membrane time constant (τ) was not statistically different in the non-running or running groups (Table 1).

**Table 1: Effects of Exercise on Basic Membrane Properties of MSSNs**

**Cm (pF)                       Rm (MΩ)                   τ (ms)**

**WT Non-Running                   **83.4±4.1                      72±7.2                         1.6±0.08

**R6/2 Non-Running                 **70.3±3.6*                    126±16*                      1.4±0.06

**WT Running                           **74.8±3.7                      79±7.7                         1.4±0.07

**R6/2 Running                         **84.1±3.6                      109±7*                        1.6±0.06

MSSNs from non-running transgenic mice had significantly reduced membrane capacitance (Cm) compared to WTs. Running alleviated the significant difference in cell membrane capacitance between the groups. In contrast, the significant increase in input resistance (Rm) in R6/2 mice was only partially alleviated by exercise. Time constant (τ) was not significantly different in non-running and running mice. Asterisks indicate the differences between WT and R6/2 mice were statistically significant.

            Spontaneous EPSCs for all experiments were recorded at -70 mV holding potential. They were mediated by AMPA/kainate glutamate receptors as they were almost completely abolished by CNQX, a non-NMDA receptor antagonist. Exercise produced a non-significant increase in the frequency of spontaneous synaptic events in WT animals in ACSF (Figure 2B, left graphs). After pharmacological isolation of EPSCs with BIC, the difference became statistically significant (p<0.05, igure 2B, middle and right graphs). BIC reduced the frequency of synaptic currents in MSSNs from non-running WT animals, but in cells from running animals the frequency of spontaneous EPSCs was increased. This increase encompassed both small (5-20 pA) and large (>20 pA) amplitude synaptic events. In contrast, in R6/2 mice the reduced levels of spontaneous synaptic currents were not enhanced by exercise in either condition. There was a trend, however, for BIC to produce a smaller decrease in the R6/2 mice in the running wheel (Figure 2B, right graph). It is tempting to speculate that the general lack of effectiveness of exercise was due to the fact that R6/2 mice displayed reduced levels of physical activity even at a very young age.

**Figure:**
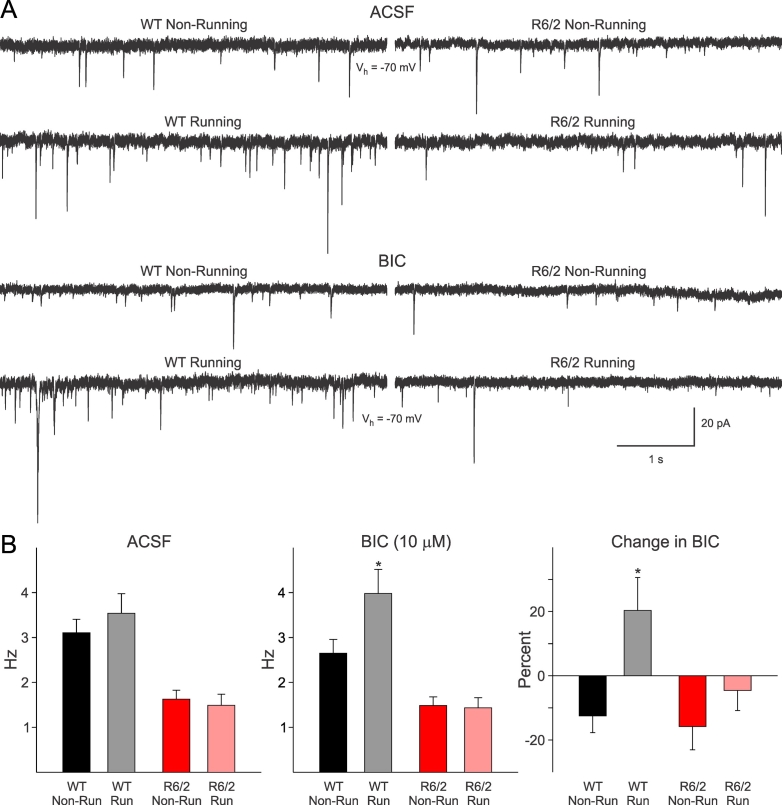

**Fig. 2: A.** Traces show spontaneous synaptic currents before (ACSF) and after bicuculline application (BIC, 10 μM) to isolate EPSCs in MSSNs from WT and R6/2 mice (V_h_=holding voltage). In both conditions, MSSNs from running WT mice displayed more spontaneous synaptic currents encompassing both small and large amplitude events. **B.** Graphs show average frequency of spontaneous EPSCs (in Hz) in MSSNs from running and non-running WT and R6/2 mice. The frequency of EPSCs was significantly reduced in R6/2 compared to WTs. After isolation of EPSCs with BIC, the average frequency of spontaneous events was significantly increased in running WT mice. While BIC reduced the number of spontaneous events in the other three groups, it significantly increased the frequency of spontaneous events in running WT mice. Significant differences (p<0.05) are indicated by asterisks.

Exercise also affected the kinetics of spontaneous EPSCs in MSSNs from running compared to non-running WT mice (Figure 3). Although decay times and half-amplitude durations were similar, the rise times were significantly faster in cells from WT running mice (Figure 3B). In cells from running R6/2 mice there also was a trend towards faster rise times, but the difference did not reach statistical significance (p=0.07). Average event amplitudes were not significantly different among groups (data not shown). 

**Figure:**
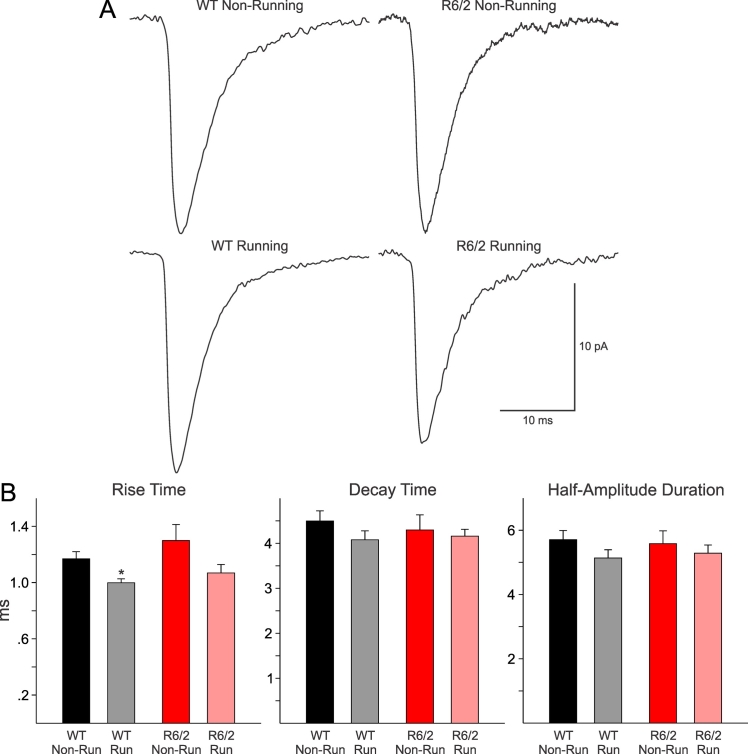

**Fig. 3: A.** Traces are averages of spontaneous EPSCs (10-50 pA) in MSSNs from running and non-running WT and R6/2 animals. No significant differences in amplitude (not shown), decay time or half-amplitude duration were found. However, the rise time was significantly shorter (p=0.005) in running compared to non-running WT mice.

BDNF protein content in non-running and running WT and R6/2 mice was determined biochemically using ELISA. At the ages examined (average 60 days), no statistically significant differences in BDNF content were found among groups. There was a small trend for higher BDNF in WT (9.99±1.2 pg/μg) compared to R6/2 (8.31±1.4 pg/μg) non-running mice and in WT (9.94±1.5 pg/μg) versus R6/2 (8.69±0.4 pg/μg) running mice but the differences were not large enough to reach statistical significance. Exercise did not change BDNF levels in WT or R6/2 mice indicating that running *per se* does not increase striatal BDNF content. 

**Adenosine: **The first adenosine receptor modulator we tested was KW 6002, a selective A_2A_ receptor-antagonist. The effects of this compound on spontaneous synaptic activity were inconsistent with cells displaying both increases and decreases in frequency (data not shown), although proportionately more cells (6/10) from R6/2 mice showed increases in frequency compared to WT cells (2/9). As A_2A_ receptors are segregated in striatum, it was possible that the lack of consistency was due to recording from different cell populations originating the direct (non-A_2A_ receptor-expressing) or indirect (A_2A_ or D2 receptor-expressing) pathways. In consequence, we also used WT mice expressing EGFP in A_2A_ adenosine or D2 dopamine receptor-containing MSSNs. Again both increases and decreases were observed. However, more cells displayed decreases in frequency, in agreement with the effects observed in unidentified WT MSSNs.

            As previous evidence indicated that the same adenosine modulators could have differential effects on NMDA receptor-mediated responses in WT and R6/2 mice [26], we tested the effects of an A_2A_ receptor agonist, CGS 21680. Indeed, this agonist produced contrasting effects in MSSNs from WT and R6/2 mice. In WT mice the agonist produced small decreases in average frequency of events whereas in cells from R6/2 mice it increased the frequency of spontaneous EPSCs (Figure 4A, B). Cumulative inter-event histograms showed that CGS 21680 renormalized EPSC frequency in that there were fewer statistically significant inter-event interval bin differences (Figure 4C).

**Figure:**
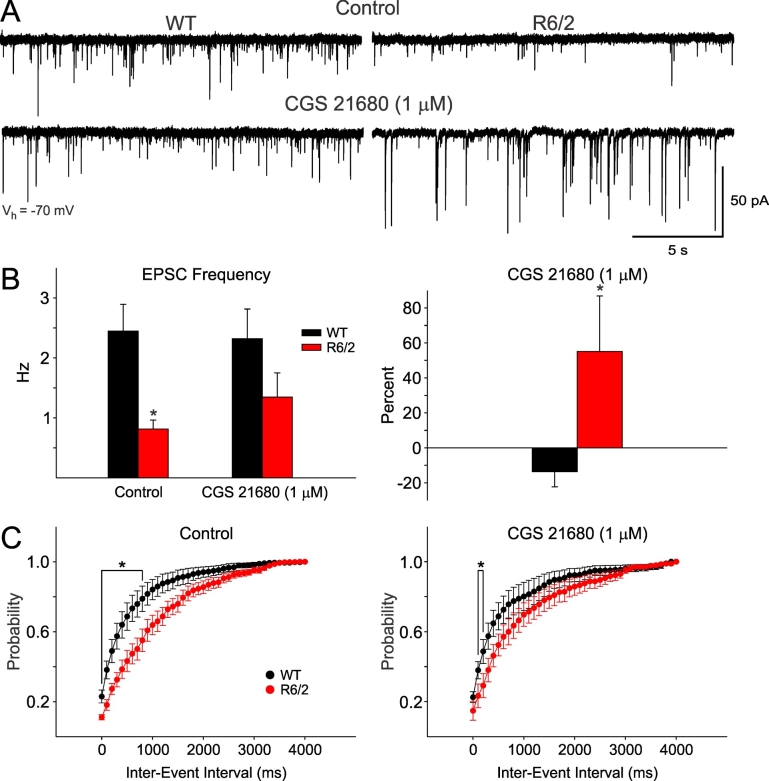

 **Fig. 4: A.** CGS 21680, a selective A_2A_ receptor agonist, produced differential effects on spontaneous EPSC frequency in WT and R6/2 mice (>60 days old). Traces show that whereas in MSSNs from WT mice the agonist produced small decreases in spontaneous synaptic events, in cells from R6/2 mice it increased the EPSC frequency. **B.** Graphs indicate significantly reduced average EPSC frequency in cells from R6/2 compared to WT mice. In cells from WT mice the agonist produced a small reduction in EPSC frequency whereas in cells from R6/2 mice it increased EPSC frequency. **C.** The cumulative inter-event histograms show that CGS 21680 renormalizes EPSC frequency in that there were fewer statistically significant inter-event bin differences between WT and R6/2 mice.

**Ampakines: **At 5-7 and 11-12 weeks, MSSNs from R6/2 mice displayed significantly reduced average frequencies of spontaneous EPSCs and mEPSCs compared to cells from WT animals [9]. mEPSC frequencies for WT and R6/2 cells were 3.5±0.4 and 2.1±0.3 Hz at 5-7 weeks and 2.6±0.3 and 1.3±0.2 Hz at 11-12 weeks, respectively. Cx614 increased the frequency and amplitude of spontaneous and mEPSCs in a dose-dependent manner (Figure 5A). This effect occurred in WT and R6/2 mice at both ages. The change in frequency in spontaneous and mEPSCs induced by a series of increasing concentrations of the ampakine is shown in Figure 5B. At 5-7 weeks there was a monotonic increase in frequency in both WT and R6/2 cells whereas at 11-12 weeks the increase in R6/2 cells was minor and reached a plateau at 100 μM concentration with no further increases at the higher concentration. 

            Examination of amplitude-frequency histograms indicated that, in spite of the increases in frequency of events, the difference between MSSNs from WT and R6/2 still remained (Figure 5C). Inter-event interval probability distributions showed that addition of Cx614 shifted the distributions to the left, indicating increased release probability (Figure 5D). However, while this shift was very pronounced in cells from WT and R6/2 mice at 40 days, in cells from 80 day R6/2 mice the differences between the distributions of events was not as pronounced. 

**Figure:**
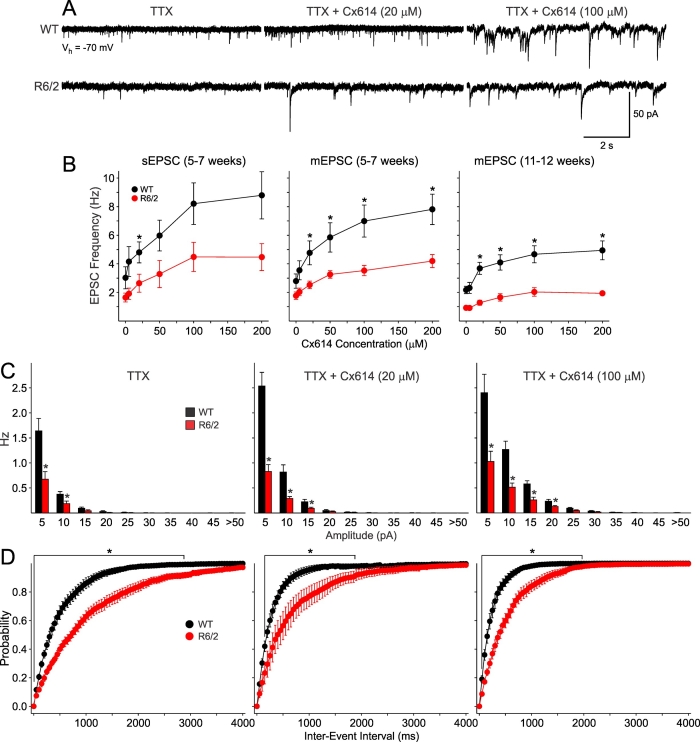

**Fig. 5: A.** Traces are examples of spontaneous mEPSCs [in the presence of TTX (1 μM)] recorded in WT and R6/2 mice (11-12 weeks of age) in the absence (left) or presence of a low (20 mM, middle) and a high concentration (100 mM, right) of Cx614. **B. **Graphs show concentration-response relationships of average frequencies of spontaneous and mEPSCs between WT and R6/2 mice at 5-7 and 11-12 weeks. Note that the increase in frequency produced at 5-7 weeks by Cx614 is less prominent in cells from both WT and R6/2 mice at 11-12 weeks of age. **C. **and **D.** Graphs show amplitude-frequency histograms (**C**) and cumulative probability distributions of inter-event intervals (**D**) of mEPSCs in MSSNs from WT and R6/2 mice before and after Cx614 (at 11-12 weeks of age). In spite of an increase in frequency of mEPSCs after Cx614, the difference between WT and R6/2 cells still remains. Line and asterisk indicate statistically significant differences between values for WT and R6/2 mice (p<0.05-0.001). 

            Cx614 also affected the amplitude and kinetics of spontaneous and mEPSCs. Amplitudes, rise and decay times, as well as half-amplitude durations increased after drug application (Figure 6A). These changes in frequency and amplitude/kinetics indicate that Cx614 could have both pre- and post-synaptic effects. In addition to the observed effects on frequency of spontaneous and mEPSCs, in a population of MSSNs from R6/2 mice Cx614, even at low concentrations, induced periodic inward currents accompanied by bursts of high-frequency synaptic activity (Figure 6B). These episodes could reflect propagation of burst firing from cortical pyramidal neurons [9], as well as increased excitability in cortical neurons in R6/2 mice [11].

**Figure:**
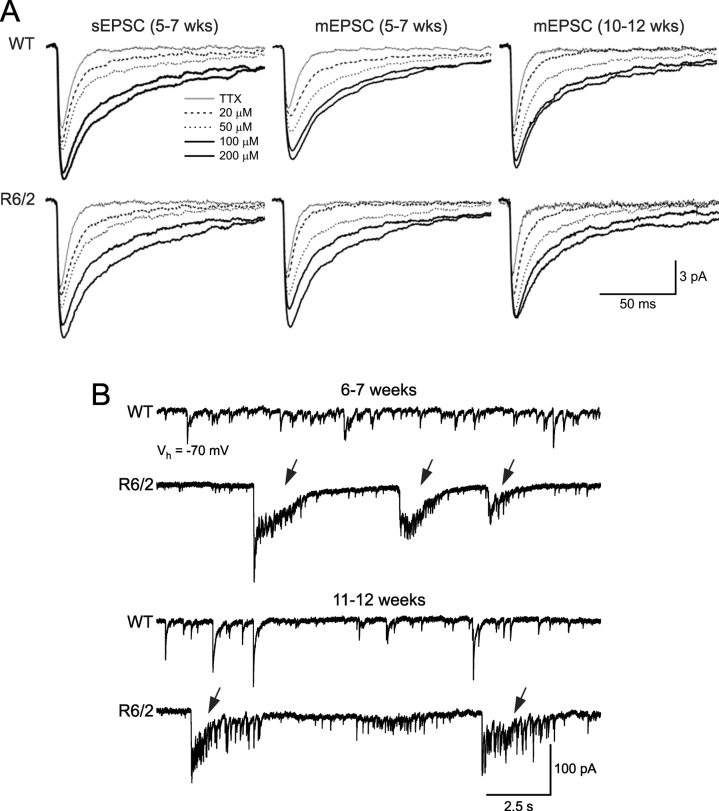

**Fig. 6: A.**  Traces are average spontaneous and mEPSCs in TTX and after bath application of increasing concentrations of Cx614. The ampakine increased the amplitude and reduced deactivation of AMPA receptors as indicated by increases in decay time and half-amplitude duration. **B.** Arrows show large, periodic inward currents were evoked by Cx614 in R6/2 but not WT mice. Representative traces recorded in the presence of Cx614 (100 mM) in MSSNs from WT and R6/2 mice at 6-7 and 11-12 weeks.

**Discussion**

A number of behavioral and pharmacological manipulations known to delay the progression of neurodegenerative disorders were tested, with the goal of rescuing the deficits in glutamatergic synaptic activity along the corticostriatal pathway in the R6/2 mouse model of HD. Each of these manipulations produced some of the desired effects, but only in a partial manner and in some cases with the potential of inducing adverse reactions. 

**Exercise:** Physical activity has shown promising effects in a number of neurodegenerative disorders including PD, AD, as well as other pathologies. When associated with an enriched environment, exercise can delay progression of the phenotype and reduce the spine loss observed in HD mouse models [23]. In WT mice, we found that exercise effectively increased synaptic activity in the striatum, indicating that the cortical flow of information is facilitated by physical activity. The fact that both small and large amplitude, action potential-dependent synaptic events in MSSNs were increased probably indicates that the firing of cortical pyramidal neurons also increases after exercise. 

            As glutamate release can also provide trophic factors, we expected that increased physical activity would rescue the progressive reduction in glutamate activity in R6/2 mice. In contrast, we found that HD mice demonstrated no significant increase in spontaneous synaptic activity. It is possible that this lack of efficacy is associated with the fact that R6/2 mice exercised significantly less that their WT counterparts. It is possible that forced, instead of voluntary, exercise could produce increased synaptic activity. However, such regimen might prove to be stressful and it is known that in R6/2 mice stress can facilitate seizure activity and precipitate death.

            Interestingly, running in R6/2 mice rescued some biophysical membrane parameters, in particular cell capacitance. The reduced membrane capacitance normally observed in MSSNs from symptomatic R6/2 mice probably reflects a loss of dendritic spines, loss of dendrites, and reduction in somatic area. Running prevented this loss of membrane. As exercise increases BDNF production [20] and BDNF can reduce the severity of HD symptoms [40], it is possible that in running R6/2 mice BDNF helps to maintain cellular integrity. BDNF determination in running and non-running mice did not reveal significant increases in protein content in soluble extracts from the striatum of R6/2 mice, consistent with a previous report [41]. However, because the ELISA assay does not differentiate between the more abundant pro-isoforms of BDNF and its mature processed form, we cannot rule out lower levels of mature BDNF that went undetected. This would be especially true for mature BDNF released at corticostriatal synapses [42], which would require more refined measurements in subcellular synaptic fractions. There is also the possibility that exercise can exert protective effects independent of BDNF and/or that other trophic factors are involved. We have previously provided evidence that the increase in input resistance could be due to a reduction of inwardly rectifying K^+^ channels [39] combined with the loss of cell membrane. The fact that exercise rescued the loss of membrane but only slightly affected input resistance could indicate that the increase in input resistance depends less on the loss of membrane than it does on reduced expression of inwardly rectifying K^+^ channels.

**Adenosine: **The findings with adenosine emphasize the complexity and multiplicity of effects induced by adenosine receptor activation or blockade, as well as the occurrence of differential effects in control and HD mice. Although some effects appear to be consistent, particularly when EGFP mice are used to identify MSSNs of the indirect pathway, the effects are not very robust and in the case of the antagonist, which has been used as a possible adjuvant in neurodegenerative disorders, the effects on synaptic activity were insignificant. The lack of robust effects of KW 6002 agrees with recent data demonstrating no change in spontaneous EPSCs using two adenosine receptor antagonists, ZM 241385 and ST 1535 [43].

            More surprising was the effect the A_2A_ receptor agonist, which effectively restored the excitatory synaptic activity in symptomatic R6/2 mice but produced little effect in WTs. This finding indicates that the progressive reduction in synaptic activity is not the passive consequence of loss of synaptic contacts but active inhibitory mechanisms also are involved. The restoration of excitatory synaptic activity by activation of A_2A_ receptors could be beneficial in the late stages of the disease. 

**Ampakines:** The efficacy of Cx614 to increase EPSC frequency and amplitude of spontaneous synaptic currents along the corticostriatal pathway was observed in both WT and R6/2 mice. This effect was age-dependent and appeared to be less robust at 11-12 weeks in both groups, but particularly in MSSNs from R6/2 mice. The increase in EPSC frequency was concentration-dependent and occurred in a range of 20 to 200 μM. This compound also affected the kinetics of the currents suggesting both pre- and post-synaptic effects.

            An unexpected finding was the exacerbation of bursting activity and the induction of large, periodic inward currents which probably reflect cortical synchronization and hyperexcitability in R6/2 MSSNs [9] [11]. Although the use of Cx614 in the R6/2 model, which also is considered a model of juvenile HD, could potentially exacerbate seizure propensity, in other models, such as knock-in mice, this compound would not involve the same risk. We have demonstrated that seizure susceptibility after administration of proconvulsant agents such as picrotoxin is not increased in knock-in mice [11]. More studies using systemic administration of ampakines *in vivo* are necessary to determine if there are potential negative effects in R6/2 mice. Thus, Cx614 could help reverse the progressive disconnection between cortex and striatum observed in HD mouse models and, at low concentrations, or in adult-onset HD, could be an effective treatment to ameliorate the motor and cognitive phenotype. 

            One limitation of the present study is that transgenic mice had variable repeat lengths. However, in our hands, decreases in spontaneous EPSCs are very similar in R6/2 mice with either ~110 or 150 CAG repeats [44] and these animals were used to examine the effects of exercise and A_2A_ receptor modulation. The behavioral and electrophysiological phenotypes in mice with ~210 CAG repeats are less severe than in the original mice with ~150 CAG repeats and are well suited to examine the effects of the ampakines. In contrast, mice with >220 CAG repeats, which were not used in the present study, display delayed development of the phenotype [45] [46].

            In conclusion, the present studies highlight some of the potential targets and treatments for a specific phenotype; the progressive disconnection between cortex and striatum, which may be responsible for late HD symptoms. Each manipulation had different success rates depending on the disease stage, suggesting that careful monitoring of patient disease stage is necessary for design of treatment regimens. In addition, our data suggest that both physical activity and polypharmacy may be indicated in order to slow the progression of HD. All of these treatments ameliorated or reversed the electrophysiological abnormalities. However, further research is required to reduce potential negative side effects of these agents.  

**Acknowledgments**

We would like to acknowledge Irene Yamazaki, Nanping Wu, Joshua L. Plotkin, Behnoud Beroukhim and Xiaoping Sun for their help in data gathering and analysis. We also thank Donna Crandall for her help with the figures.****

**Funding**

This study was supported by grants from the USPHS (NS41574), the High Q Foundation, the Cure HD Initiative and the Hereditary Disease Foundation. 

**Competing Interests**

The authors have declared that no competing interests exist.****
